# Ulduz Maschaykh: The changing image of affordable housing: design, gentrification and community in Canada and Europe: Ashgate, 2015, ISBN: 978-1-4724-3779-2

**DOI:** 10.1007/s10901-016-9503-8

**Published:** 2016-02-13

**Authors:** Brian Doucet

**Affiliations:** Erasmus University College, Nieuwemarkt 1A, 3011 HP Rotterdam, The Netherlands

This book takes an architectural perspective towards understanding the challenge of housing affordability in cities. It is primarily focused on British Columbia, Canada, with a few brief case studies in Europe. The author’s main argument is that: ‘[A]rchitecture can play a pivotal role in diminishing social imbalances and giving people from a broader range of backgrounds a sense of identity and belonging. In this regards, buildings can influence wider social and political changes’ (p. 9).

Chapter one provides a thorough review of the main literature on gentrification. Maschaykh describes four theories in good detail: Smith’s rent-gap, Florida’s creative class, David Ley’s middle-class aesthetic and the trend towards mixed-income communities. One of the best parts of this book is when she provides cultural references from television, books and movies to illustrate each theory. What is missing, however, is an analysis or interpretation of the relationship between gentrification and the lack of affordable housing. The author’s position on gentrification is never fully articulated and chapter one reviews, rather than frames the author’s position in the literature. She writes of gentrification: ‘[W]hen a city’s value increases and draws wealthy residents and investors into rundown neighbourhoods, these enclaves experience a process of revitalization that is known as ‘gentrification.’ Architecture plays an important part in the gentrification process. This is most evident in buildings that stand out from the rest because of their history’ (p. 2). Exactly what role architecture plays is not clear. Does it stimulate or reduce gentrification and if so, how? Is this good for cities; if so why? To make a strong contribution to the gentrification debate, such questions needed further analysis and elaboration.

Empirically, the book deals with some insightful case studies. Part one focuses on Europe, with chapter two examining early twentieth Century affordable housing in Vienna and Germany. Examples such as Vienna’s Karl-Marx-Hof are described in detail. In chapter three, the focus shifts towards contemporary ‘super-gentrification’ in Berlin. Maschaykh departs from her architectural perspective and argues that current pressures on affordable housing in Berlin are a result of both population growth and laws passed by the Berlin government to encourage the development of luxury real estate projects at the expense of housing for lower and middle-classes.

In the second part of the book, attention turns towards Canada and its approach to affordable architecture. The opening text to this section provides a general, if somewhat patchy overview history of social housing in Canada.

The case studies in chapters four through six are interesting and described in great detail. But they help to underscore the ambiguous relationship between gentrification and affordable housing presented by the author. Several examples are described as being a catalyst for gentrification. But many scholars argue that gentrification produces greater affordability issues in cities (Lees et al. 2008). Critical perspectives on gentrification are not fully conceptually and theoretically countered in this book and the author tends to equate gentrification with increases in affordable housing opportunities, particularly for young, professional households. While the case studies she describes may give these households more opportunities and contribute to neighbourhood ‘revitalisation’, there is a flip-side of displacement of poorer households which is not adequately incorporated into the book’s analysis and conclusions.

Chapter four deals with small-scale developments and historic preservation in Victoria. The Cornerstone initiative, for example, is celebrated as an example of affordable housing which preserves a historic building. It has a café, which has spearheaded gentrification in the neighbourhood. She writes: ‘This makes it an unusual example of gentrification, as an affordable housing project actually led to the revitalisation of the neighbourhood.’ (p. 80). Despite offering a handful of units of affordable housing, the reader is left wondering if this building actually contributes to gentrification, and therefore wider issues of affordability throughout the neighbourhood? (see, for example, August 2008).

Chapter five primarily describes the transformation of single-room occupancy (SRO) hotels into affordable apartments for young professionals in Vancouver’s Downtown Eastside. She uses several examples of buildings which have made this transition, including The Burns Block whose transformation is described as: ‘[T]he risky projected turned out very successfully: a run-down heritage building was rescued from further deterioration, young tenants were given affordable and liveable dwelling options in the city centre … and the corner of Carrall and West Hastings is slowly becoming a thriving part of the neighbourhood’ (p. 104). Again, while the neighbourhood is reviving, the displacement of the tenants in the SROs and what the loss of this type of housing means for its former inhabitants is not discussed in detail (see, Slater 2004). The final chapter, titled ‘the Poor, the Lost and the Forgotten,’ focus on examples of affordable housing where gentrification is not present, such as seniors housing in Kelowna.

The structure and approach of this book are at times confusing. The primary aims and research questions are not clearly spelled out and the introduction jumps between several distinct and disparate topics where the reader is left wondering what the major links are. In some cases, this confusion is made more difficult by the writing style which jumps between topics rather abruptly.

The selection of British Columbia is a good choice for a case study and the inclusion of smaller cities is most welcome in a body of literature that primarily focuses on larger cities. However, the rationale and selection criteria for the international comparison are not thoroughly explained. The comparison with Europe is out of place. While Central Europe is also an insightful place to study affordable housing, the nature of the trans-Atlantic comparison is rather vague and ambiguous. The cases cover different time periods, topics and types of developments at different geographic scales. For example, much of Chapter three is spent discussing ‘super-gentrification’ in Prenzlauerberg in Berlin, yet no such comparison is done in British Columbia, which is surprising considering the extent of gentrification in Vancouver.

Maschaykh states that architecture can influence social and political changes. While the case studies are interesting and detailed, they lack a sound theoretical grounding and conceptual rigour to convincingly address this issue. Part of this has to do with the author’s own ambiguity regarding the concepts of affordable housing, the nature of gentrification and the role of class change and displacement. There is also no strong conclusion to the book; a discussion on how the empirical case studies relate to the four theories of gentrification summarised in chapter one would have given the book focus, positing and made a stronger contribution to academic debates.
